# Characterization of Highly Filled PP/Graphite Composites for Adhesive Joining in Fuel Cell Applications

**DOI:** 10.3390/polym11030462

**Published:** 2019-03-11

**Authors:** Piotr Rzeczkowski, Beate Krause, Petra Pötschke

**Affiliations:** Leibniz-Institut für Polymerforschung Dresden e.V., Hohe Str. 6, 01069 Dresden, Germany; rzeczkowski@ipfdd.de (P.R.); krause-beate@ipfdd.de (B.K.)

**Keywords:** polypropylene/graphite composites, thermal conductivity, electrical conductivity, mechanical properties, adhesive joining, bipolar plates, fuel cells

## Abstract

In order to evaluate the suitability of graphite composite materials for use as bipolar plates in fuel cells, polypropylene (PP) was melt compounded with expanded graphite as conductive filler to form composites with different filler contents of 10–80 wt %. Electrical resistivity, thermal conductivity, and mechanical properties were measured and evaluated as a function of filler content. The electrical and thermal conductivities increased with filler content. Tensile and flexural strengths decreased with the incorporation of expanded graphite in PP. With higher graphite contents, however, both strength values remained more or less unchanged and were below the values of pure PP. Young’s-modulus and flexural modulus increased almost linearly with increasing filler content. The results of the thermogravimetric analysis confirmed the actual filler content in the composite materials. In order to evaluate the wettability and suitability for adhesive joining of graphite composites, contact angle measurements were conducted and surface tensions of composite surfaces were calculated. The results showed a significant increase in the surface tension of graphite composites with increasing filler content. Furthermore, graphite composites were adhesively joined and the strength of the joints was evaluated in the lap-shear test. Increasing filler content in the substrate material resulted in higher tensile lap-shear strength. Additionally, the influence of surface treatment (plasma and chemical) on surface tension and tensile lap-shear strength was investigated. The surface treatment led to a significant improvement of both properties.

## 1. Introduction

Fuel cells seem to be promising energy converters, which are more economically and environmentally friendly than common energy converters, e.g., heat engines. The interest in fuel cells has increased in recent years and their development is the aim of many research projects. 

A fuel cell consists of two bipolar plates, which are firmly joined together providing efficient and reliable power generation. The most commonly used materials for the manufacturing of fuel cells are metals, due to their high electrical and thermal conductivity and better processability than polymer materials. On the other hand, metals have much higher density than polymers, which leads to a high weight of fuel cell stacks, and they are susceptible to corrosion, which can lead to problems in fuel cell operation. Polymers filled with conductive fillers can be considered as very good alternative to metals. Polymer composites can guarantee sufficient electrical and thermal conductivity and good processability of bipolar plates with low weight of the fuel cell and can successfully replace metals in fuel cell applications in the future.

Fuel cells are devices that are able to convert chemical energy into electricity with a relatively high degree of efficiency. This is a big advantage compared to heat engines (e.g., combustion engines), which first convert chemical energy into mechanical energy and then into electricity by means of a generator, which leads to major energy losses. But their high manufacturing costs and high weight have so far restricted their application in industry and in households [1]. Proton-exchange membrane (PEM) fuel cells are particularly interesting for the automotive industry and portable energy generators, due to their high power density, very low environmental pollution and low operating temperature (max. 100 °C) [2]. The heart of the PEM fuel cell is the Membrane Electrode Assembly (MEA), which is pressed together from both sides by two bipolar plates forming a single fuel cell. Several single fuel cells stacked one behind to the other form a fuel cell stack. A bipolar plate has following functions in the fuel cell [3]: distribution of the propellants within the fuel cell, transfer of electrons from one cell to the adjacent cell, removal of reaction water and heat dissipation.

Due to the very low weight of MEA, the weight of bipolar plates largely determines the total weight of the fuel cell (around 80%) and its final price. Carbon based polymer composites gain in significance due to good electrical and thermal conductivities, sufficient mechanical properties, and a significantly lower weight compared to metals. Polymer composites can easily be manufactured using common processing methods such as injection molding or hot pressing. Injection molding is very well suited for mass production, but can be limited by higher filler contents (approx. 60–80 wt %) in the polymer composite. Composites with higher filler contents can be manufactured by hot pressing, but require longer cycling times than injection molding. For this reason, an optimum composition of graphite composites for the processing of bipolar plates should be developed.

The overall requirements for bipolar plates in PEM fuel cells are listed in Table 1.

One of the biggest problems with fuel cells is a possible leakage of propellants. Therefore, a proper and secure seal between two adjacent bipolar plates must be ensured. Such a seal must ensure water- and gas-tight operating of the fuel cell. The most commonly used seals are rubber gaskets with special gaps for feeding channels in bipolar plates. This method involves time-consuming assembly (hand lay-up of rubber gaskets) and requires a high contact pressure to ensure sufficient tightness, which can be critical with brittle graphite composites. Sometimes even a higher contact pressure is not sufficient, which leads to the leakage of propellants during operations of the fuel cell. Adhesive joining can be considered as a good alternative sealing method in fuel cells. The chemical bonds between adhesive and substrate can create very tight bonding between the both bipolar plates, preventing propellant leakage. The further advantages over the conventional sealing method are the automated application of adhesive (e.g., robot arm) and no high contact pressure during the entire operations of the fuel cell [4,5].

Polymer composites filled with carbon-based fillers have already been extensively investigated in many research works and publications. In reference [6], the electrical conductivity of polymer composites made of polypropylene (PP), polyaniline (PANi), synthetic graphite or carbon black, and their processing methods (melt mixing or solution mixing) were investigated. Depending on the filler content or material composition (matrix composition or filler type), a significant increase in electrical conductivity compared to the neat polymer could be observed. The highest electrical conductivity of 36.4 S/cm was achieved for a PP-based composite consisting of 55 wt % graphite and 25 wt % carbon black prepared by melt mixing. An electrical conductivity of 23.2 S/cm was measured for a PP-based composite with 80 wt % of graphite (solution mixing). In [7] the compounding of PP/EPDM matrix with carbon black and different kinds of graphite led to a significant increase of thermal conductivity and decrease of electrical resistivity of obtained composite materials. Using the same type of expanded graphite as in this paper, a thermal conductivity of 12.2 W/m·K for PP/EPDM + 60 wt % graphite and 15.5 W/m·K for PP/EPDM + 80 wt % of graphite was achieved. In reference [8], highly filled polymer composites with a constant filler content of 78 wt % graphite with different graphite grades and different particles sizes were prepared by injection or compression molding. The electrical conductivity values were dependent on the graphite type and the processing method. The highest electrical conductivity of 20.6 S/cm was observed for a composite of flake-like graphite with a particle size of 5 µm. In reference [9], polypropylene-based polymer composites filled with 70 wt % of various graphite types were investigated. With expanded graphite, a thermal conductivity of around 8.5 and 22 W/m·K were reached, depending on the graphite type. For composites with 80 wt % of graphite, flexural strength of about 50 MPa and flexural modulus of about 15 GPa were achieved. In a dissertation [10], polypropylene composites with carbon-based filler were investigated with regard to their application in fuel cells. For composites with 80 wt % synthetic graphite, a thermal conductivity of 6.0 W/m·K and an electrical conductivity of 11.1 S/cm were obtained. In reference [11], an attempt was made to develop a bipolar plate consisting of a PP/graphite housing and stainless steel core, on which contact resistance and flexural strength were measured. To reduce the contact resistance, carbon nanotubes were applied directly to the steel core, reducing contact resistance by about 40%. At the same time, flexural strength of the composite decreased with increasing CNT content. The thermal and electrical conductivity of graphite-filled PP-based composites was investigated in reference [12]. At a filler content of 20 wt %, a thermal conductivity of 1.1 W/m·K and an electrical conductivity of about 1 S/cm were achieved. In reference [13], the thermal conductivity of PP-based composites with increasing graphite content (up to 40 wt %) was investigated. The highest thermal conductivities were achieved at 40 wt % graphite, whereby an ordered distribution of the graphite flakes in the polymer matrix led to a higher thermal conductivity (5.4 W/m·K) than the random distribution (1.6 W/m·K). Changes in the mechanical properties of PP-based composites with different graphite contents (10, 30 and 50 wt %) were investigated in [14]. The results showed that tensile strength reached 17.4 MPa and Young’s modulus 5538 MPa at 50 wt % graphite. In references [15,16,17], there are some reviews on the properties of polymer composites filled with carbon-based fillers for fuel cell applications. 

The adhesive joining of graphite composites for fuel cells application has not been fully investigated and only a few reports have been found. In references [18,19], shear strength after high temperature treatment of adhesively joined graphite substrates with phenol resin-based adhesives with different fillers (boron carbide, silicon dioxide) was investigated. After treatment up to 200 °C, adhesively joined parts failed within graphite substrate. Another adhesive for joining graphite parts, based on silicon or graphite powder and polymeric resin was developed in patent [20]. The patent [21] reports on microencapsulated adhesive for joining bipolar plates in fuel cells. Finally, reference [22] reported on an attempt to join carbon fiber/epoxy bipolar plates with a silicone adhesive. Tensile and tensile lap-shear strengths of adhesively joined fuel cell polymer membranes or carbon fiber/epoxy composites were measured. Both strengths of adhesively joined carbon fiber/epoxy composites were below 1 MPa and were lower than those of joined polymer membranes. In reference [23], surface tensions of highly filled PP/EPDM/graphite composites were investigated, with a significant increase in surface tension with high filler content in composites (51.3 mN/m for PP/EPDM + 80 wt % of graphite) compared to neat PP (29.8 mN/m). The plasma treatment of the surface resulted in a significantly higher surface polarity of the graphite composites. 

In the present study, PP composites with different contents of expanded graphite (10–80 wt %) were compounded by twin-screw extrusion and investigated for their application in fuel cells. The aim was to investigate the influence of filler content in the polymer matrix on the mechanical, thermal, and electrical properties of the composite materials and to compare these with the target values for fuel cell applications. In order to estimate the suitability of graphite composite for adhesive joining, the surface tension and its changes after surface treatment (plasma and chemical) were studied. In addition, mechanical properties of adhesively joined graphite composites were measured in the tensile lap-shear test. A surface treatment (plasma and chemical) was also performed to improve tensile lap-shear strength of joined materials.

## 2. Materials and Methods

### 2.1. Materials

Polypropylene (PP) SABIC^®^ 579S (Sabic, Riyadh, Saudi Arabia) with a melt flow rate of 47 g/10 min at 260 °C/2.16 kg was used as polymer matrix for the preparation of the composites.

For the electrically and thermally conductive filler, expanded graphite powder Sigratherm^®^ GFG600 (SGL Carbon, Meitingen, Germany) was used. The particle size was determined as *x*_10_ = 106 µm, *x*_50_ = 269 µm, *x*_90_ = 395 µm and *x*_99_ = 444 µm (mean value of three measurements) [7]. 

For the joining of graphite composites, a commercially available epoxy-based adhesive system ScotchWeld DP 490 (3M Deutschland, Neuss, Germany) was used.

### 2.2. Materials Processing

Polymer nanocomposites with increasing filler content from 10 to 80 wt % were melt mixed in a Berstorff ZE 25 twin-screw extruder (screw length 48D, T = 180–200 °C, 10 kg/h) (KraussMaffei Berstorff GmbH, Hannover, Germany) with two side feeders for feeding the filler. Table 2 lists the process parameters for melt mixing. For composite materials with 60 wt % and 80 wt %, the extrusion of homogeneous materials had to be adapted so that the screws could take up the necessary amount of graphite. This was only possible with an increased screw speed and polymer melt temperature (230 °C and 300 °C, respectively) compared to the other materials. Due to the higher filler content, the viscosity of the polymer melt and thus the pressure at the nozzle increases significantly. Different processing parameters cause different shear conditions in the polymer composite melt and can lead to a different dispersion and distribution of both components in the composite. Unfortunately, a meaningful characterization of the filler distribution in such highly filled composites is not possible due to the large particle size and the high volume fraction. Thermogravimetric analysis (TGA) confirms the actual filler content in composite materials (Table 2). 

The injection molding of plates 80 × 80 × 2 mm^3^ was carried out with the Allrounder 420C 1000-25 (ARBURG, Loßburg, Germany) at a melt temperature of 202 °C, a mold temperature of 55 °C, an injection pressure of 190 bar, and an injection speed of 25 mm/s.

### 2.3. Joining of the Samples

Strips with dimensions of 25 × 80 mm^2^ were cut from the injection molded plates and milled to a thickness of 1.8 mm. These samples were adhesively joined to single lap joints with an overlap length of 12.5 mm and cured for 7 days at room temperature. The expected thickness of the bondline was 0.2 mm, but actual thickness of the bondline of the adhesively joined samples was between 0.15 mm and 0.3 mm. Samples with pre-treated surfaces were joined in the same manner within 3 h after treatment.

### 2.4. Surface Pre-Treatment

The pre-treatment was carried out on the injection molded samples with milled surfaces. Plasma treatment was conducted in the vacuum plasma device TePla 440G (PVA TePla, Wettenburg, Germany) using oxygen as propellant with the following set-up parameters: gas flow rate 10 cm^3^/min, pressure 0.23 mbar, and treatment time 60 s. Chemical treatment was carried out by immersing the samples in a sulfuric acid-based solvent at 70 °C for 2 min [24]. Afterwards, the chemical treatment samples were flushed with distilled water and dried.

### 2.5. Characterization Methods

The agglomerate size distribution of the graphite powders was determined by laser diffraction with a HELOS/BF particle size analyzer coupled to a RODOS dry dispersion unit (Sympatec GmbH, Clausthal-Zellerfeld, Germany) and an ASPIROS micro dosing module (Sympatec GmbH, Clausthal-Zellerfeld, Germany). The measuring range is 4.5–875 μm.

The thermal conductivity (λ) of graphite composites was measured at 25 °C using the laser flash method with an LFA 447 device (Netzsch GmbH, Selb, Germany). The samples for thermal conductivity measurements were compression molded with a hot press PW40EH (240 °C, 50 kN, 2 min) (Paul-Otto Weber, Remshalden, Germany) into cylindrical shape (diameter 12.7 mm, thickness 2 mm). 

The electrical volume resistivity (*ρ*) of graphite composites was measured at 25 °C using the electrometer E6517A (Keithley Instruments, Cleveland, OH, USA) and the multimeter DMM2001 (Keithley Instruments, Cleveland, OH, USA). The samples for the electrical volume resistivity measurements were compression molded with the same pressing machine and the same process parameters as for the thermal conductivity, but in plates with thicknesses between 0.3 mm and 0.8 mm. The samples were cut to strips with width of 3 mm and measured on both sides. The electrical conductivity was calculated as the reciprocal value of the electrical resistivity obtained:

(1) $$\sigma =\frac{1}{\rho }.$$

The TGA analysis of the graphite composites was performed with a TGA Q5000 (TA Instruments, New Castle, DE, USA) between 25 °C and 800 °C under nitrogen atmosphere at a scan rate of 10 K/min. The same samples as for thermal conductivity were used for the TGA measurements. 

The mechanical properties of the graphite composites were investigated using the Zwick Roell 1456 universal testing machine (tensile test) and Zwick Roell Z 2.5 (flexural test) (both Zwick Roell Group, Ulm, Germany). The tensile and flexural strengths, E-modulus and flexural modulus, and tensile strain at break and the flexural strain at maximum stress were determined. As PP showed high plasticity of PP and missing fracture of the specimens in the flexural tests, the strain at maximum stress were selected for comparison. The samples for the mechanical tests were produced by injection molding in the standard specimen shapes recommended in DIN EN ISO 527-2/1A (tensile test) and DIN EN ISO 178 (flexural test). 

The mechanical properties of the adhesively joined composites were investigated in the lap-shear test with the Zwick Roell 1456 universal testing machine (Zwick Roell Group, Ulm, Germany). 

The dynamic contact angle (θ) was measured on the surface of graphite composites using the OCA 35 XL device (Data Physics, Filderstadt, Germany). Three liquids with different polarities were used: distilled water, 1,5-pentanediol, and diiodmethane (Table 3). The advancing contact angle was calculated as the mean of five drops of each liquid on the composite surface. The surface tension (*σ_s_*) and its dispersive (*σ_s_^D^*) and polar (*σ_s_^P^*) parts of the graphite composite surfaces were calculated according to the model of Owens-Wendt-Rabel-Kaelble (OWRK):

(2) $$\sigma_{sl}=\sigma_{s}+\sigma_{l}-2\left(\sqrt{\sigma_{s}{}^{D}+\sigma_{l}{}^{D}}+\sqrt{\sigma_{s}{}^{P}+\sigma_{l}{}^{P}}\right)$$

and the Young’s equation:

(3) $$\sigma_{s}=\sigma_{sl}+\sigma_{l}\cdot \cos\theta$$

The contact angle of the samples with pre-treated surfaces was measured on the same day as treatment process in the same way.

The surface roughness was measured with the confocal 3D microscope µsurf expert (Nanofocus AG, Oberhausen, Germany). The profile factor (*R_a_*) and the 3D-surface factor (*S_dr_*) were determined to characterize the surface roughness.

Contact angle and roughness were measured on 80 × 80 × 2 mm^3^ injection molded plates whose surfaces were milled to remove a thin upper polymer layer on the surface.

## 3. Results and Discussion

### 3.1. Mechanical Properties of Graphite Composites

**Figure 1:**
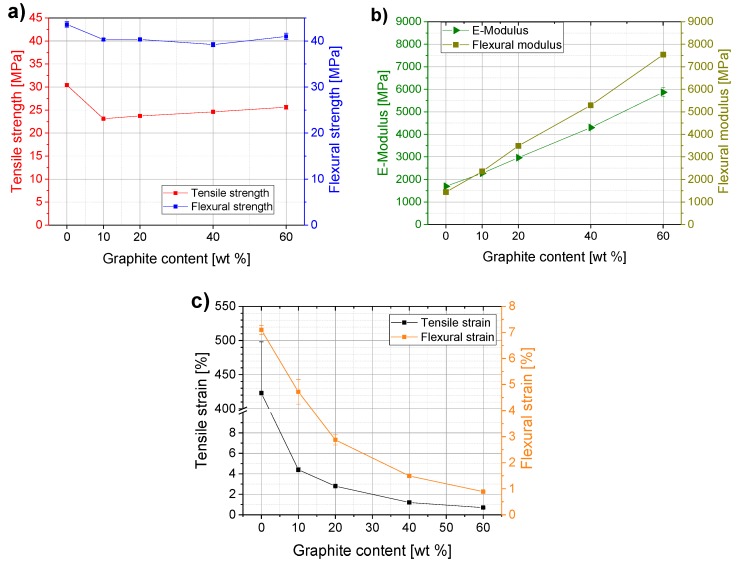
Mechanical properties of PP/graphite composites depending on filler content: (**a**) tensile and flexural strengths; (**b**) E-Modulus and flexural modulus; (**c**) tensile strain at break and flexural strain at maximal stress.

The mechanical properties could only be investigated for PP composites up to 60 wt %, since injection molding of composite with 80 wt % was not possible. The addition of higher amounts of expanded graphite to the polymer matrix results in significant changes in the mechanical properties. A similar behavior in tensile and flexural strengths could be observed (Figure 1a). An addition of 10 wt % of graphite leads to a 25% lower tensile strength compared to neat PP. Composites with higher filler contents up to 60 wt % show a slight linear increase of tensile strength, compared to PP/10. The maximum achieved value of 25 MPa at 60 wt % graphite loading does not yet meet the requirement specified in Table 1 (>41 MPa). The flexural strength decreases by about 10% after addition of 10 wt % graphite and remains at this level up to PP/60. All flexural strength values of this study meet the requirement of >25 MPa specified in Table 1. Flexural and E-moduli increase linearly with increasing filler content, indicating that graphite reinforced composites have higher stiffness (Figure 1b). Both tensile strain at break and flexural strain at maximum stress show a significant decrease after the addition of graphite to the polypropylene matrix. Already after the addition of 10 wt % graphite, the tensile strain at break decreased enormously from 420% to 4% and decreased further at higher filler contents down to 0.7% at 60 wt % graphite. The changes in flexural strain at maximum stress are more gradual and not as drastic as for the tensile strain at break (Figure 1c).

Both composite components, polypropylene and graphite, have very different mechanical properties. PP is a polymer with very high plasticity and graphite is a very brittle material. This is the main reason why compounding both materials results in lower plasticity and higher stiffness and brittleness than the original properties of pure polymer. It appears that the composite materials are more likely to adopt the mechanical properties of graphite than those of polypropylene. This is particularly evident in the tensile and flexural stiffness (both moduli) as well as in the tensile strain at break, where the values already change significantly after the addition of only 10 wt %. 

Regarding the general trends, the results obtained here are in line with the previous work on PP-based composites. In general, a linear increase in Young’s modulus (E-modulus) and flexural modulus is observed by adding stiffer fillers. For the tensile strength ref. [26] reports a slight increase of maximum 2 MPa and with the maximum added content of 20 wt % graphite values comparable to pure PP (31 MPa). In reference [14], a decrease in tensile strength was reported for PP-composites when the graphite content was increased from 10 wt % to 30 wt % and 50 wt %. Contrary to our results that the tensile strength remains at a constant value of approximately 25 MPa between 10 wt % and 60 wt % load, this study reports a significant decrease of approximately 40% between 10 wt % and 50 wt % graphite and values of approximately 17 MPa for PP/50 wt % graphite. The tensile strain at break decreases in a similar way as in our study. 

### 3.2. Thermal Conductivity of Graphite Composites

**Figure 2:**
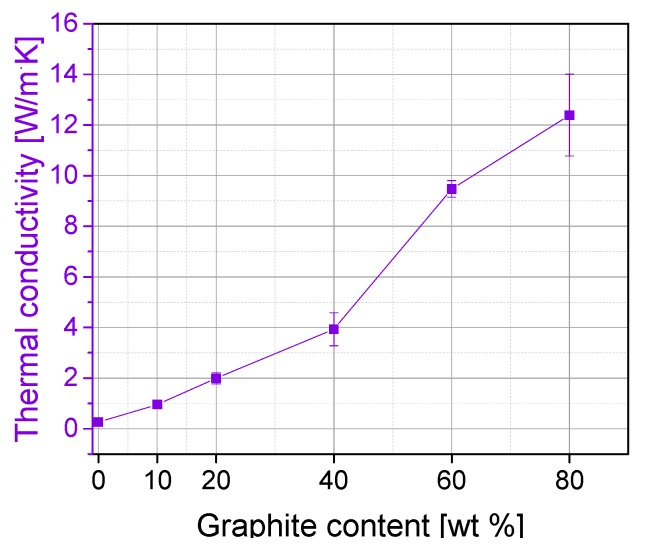
Thermal conductivity of PP/graphite composites.

Figure 2 shows the results of thermal conductivity measurements of graphite composites with different filler amounts. With the addition of graphite particles a gradual increase of the thermal conductivity of the composite materials can be observed. After an almost linear increase of up to 40 wt % graphite, this is clearly more pronounced at higher filler contents. At 80 wt % graphite, a thermal conductivity value of 12.4 W/m·K is achieved, which fulfils the requirement according to Table 1. 

However, the values are lower compared to those reported in reference [7] for PP/EPDM-based composites, where slightly higher thermal conductivities of 12.2 W/m·K and 15.5 W/m·K were found for composites with 60 and 80 wt % expanded graphite, respectively. Comparing the thermal conductivity at 20 wt % graphite, the values obtained are higher than those given in references [12,13], where thermal conductivities of 1.0 W/m·K and 1.5 W/m·K respectively were achieved for PP composites. 

### 3.3. Electrical Conductivity of Graphite Composites

**Figure 3:**
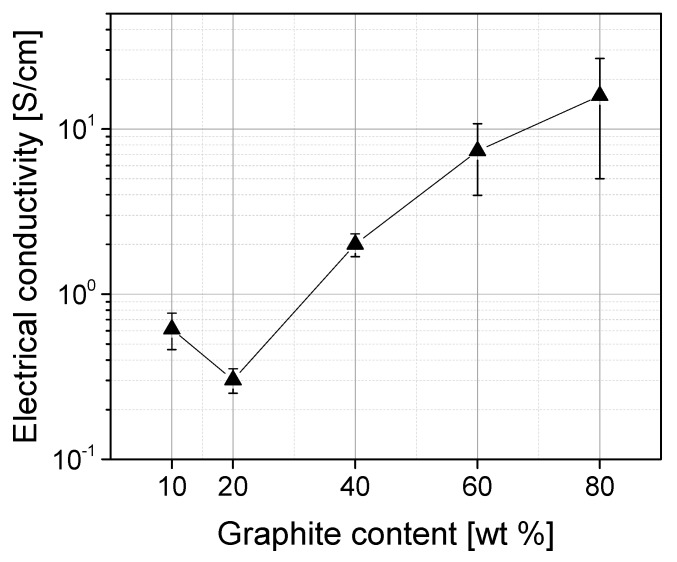
Electrical conductivity of PP/graphite composites.

The electrical conductivity also shows the tendency to increase with the filler content (Figure 3). Even the sample with 10 wt % graphite is electrically percolated and has a conductivity value of approx. 0.6 S/cm. With 20 wt % graphite there is a slight decrease, followed by an almost linear increase of the conductivity values up to 15.9 S/cm for PP composites with 80 wt % graphite. However, this value is still clearly below the target value according to Table 1. 

The values achieved are in the range of those from reference [6] where a PP/synthetic graphite composite showed a similarly increasing behavior of the electrical conductivity with the filler content and the following highest values were achieved for materials with 80 wt % graphite: 7.0 S/cm (melt mixing) and 23.2 S/cm (solution mixing). The electrical conductivity results reported in reference [8] of PP-based composite materials with 78 wt % graphite were in the same conductivity range: values between 5 S/cm and 22 S/cm were measured, depending on the shape or particle size of the graphite and the measurement parameters (contact pressure). The achieved electrical conductivity values of this work are better than those reported in [7], where electrical conductivities of 1.3 S/cm and 5.3 S/cm were achieved for PP/EPDM-based composites with 60 wt % and 80 wt % of the same expanded graphite as in this study. Obviously the EPDM addition improves the processing behavior of the composites, but has a slightly negative effect on the electrical conductivity.

### 3.4. Contact Angle and Surface Tension

Dynamic contact angle measurements were performed to assess the surface tension and its polar and dispersive parts. The surface tension of materials is generally very important for adhesive joining [27]. Therefore, the surface tension of the graphite composites was calculated to verify the suitability for adhesive joining. A higher surface tension of the substrate has a positive effect on the wettability and adhesion between substrate and adhesive and improves the aging properties of the joint [28]. It is expected that a composite with higher filler content may be more suitable for adhesive bonding than pure PP or a composite with lower filler content. The polarity of the surface has a significant influence on both wettability and adhesion. Polar molecules on the substrate surface can produce stronger chemical bonds with an adhesive, allowing a stronger and more durable bond.

**Figure 4:**
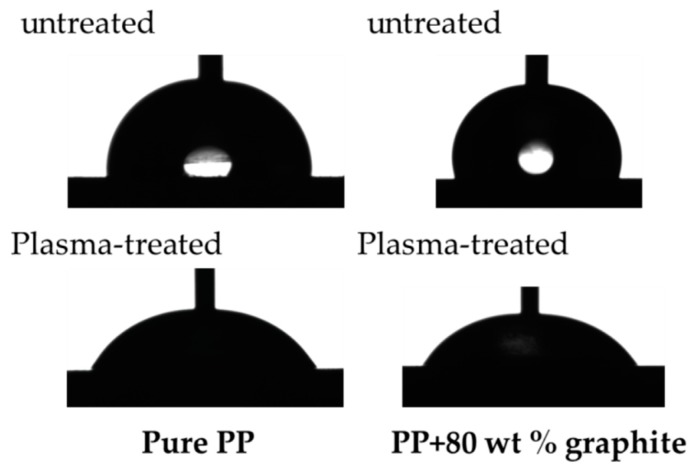
Water contact angle on untreated and plasma-treated surfaces of pure PP (**left**) and PP/80 (**right**).

The drop profiles shown in Figure 4 demonstrate that the PP-based materials have a very high water contact angle. While the untreated surfaces of pure PP shows a value of 85.6° ± 5.7°, PP/80 has a slightly higher value of 101.1° ± 3.0°. The high contact angles are caused by different polarities of the water and both substrate surfaces. Water has a high polar part of surface tension (see Table 3) and therefore produces a high contact angle with non-polar surfaces (such as pure PP and PP/80). If the polarities of substrate and liquid are less different between their values, the contact angle is smaller, resulting in better wettability of the surface. Comparably high contact angle values were reported in reference [28], where a 105° contact angle with water was measured on a surface of an epoxy-based composite material containing 80 wt % graphite. 

**Figure 5:**
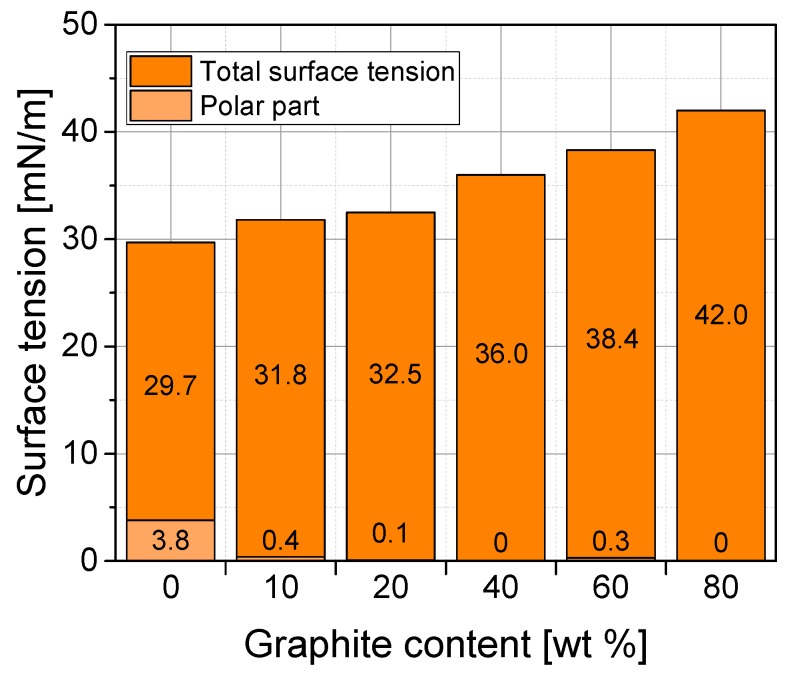
Surface tension and its polar part of PP/graphite composites with different graphite content.

**Figure 6:**
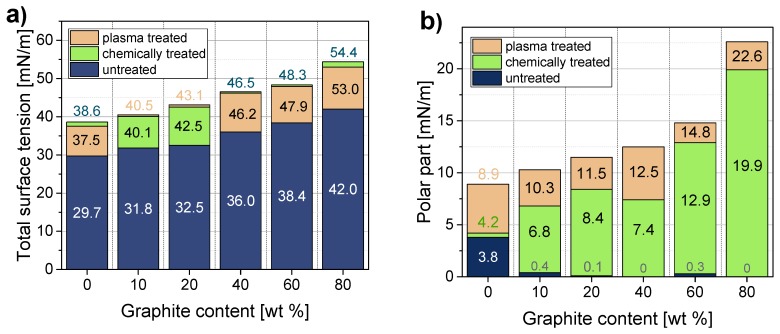
Effect of surface treatment on (**a**) total surface tension and (**b**) its polar part of pure PP and PP/graphite composites.

The surface tension of the graphite composites was calculated using the obtained contact angles and Equations (2) and (3). This calculation model makes it possible to determine not only the total surface tension, but also its polar and dispersive parts. Figure 5 shows changes of the surface tension and its polar parts at different filler contents. As explained in reference [27], if polyethylene surfaces are to be bonded, the surface tension of this non-polar material must be increased to at least 48–52 mN/m. Since polyethylene and polypropylene have very similar surface properties, these values can also be regarded as target values for PP. A significant gradual increase in surface tension can be observed as the graphite content increases. The surface tension of pure PP (29.7 mN/m) is increased to 141% after the addition of 80 wt % graphite (42.0 mN/m). Nevertheless, the surfaces of all graphite composites remain non-polar and exhibit very low polar parts of surface tension, which are even lower than the measured polar part of pure PP. Since the polar part does not change with increasing filler content in the composites, the increase in the total surface tension is caused by an increasing dispersive part. In order to increase the surface tension and polarity of the graphite composites, two pre-treatment methods (plasma and chemical) were used, which are well established in polymer surface treatment [24]. The contact angles were measured on the pre-treated surfaces and the surface tensions including their polar parts were calculated (see Figure 5 significant reduction of the contact angle after plasma treatment was observed for pure PP and PP/80 (see Figure 4, bottom line). A distinct increase in the surface tensions of all materials after both, plasma and chemical treatment, was observed (Figure 6a). Based on these results, it is not possible to specify which treatment method is more suitable, as the increase in surface tension for all materials is in the same range (about 10 mN/m) for both methods. Figure 6b shows that surface pre-treatment leads to a significant increase in polarities for all materials. It is interesting to note that a higher graphite content in the composite leads to a higher polarity increase after surface treatment. This becomes particularly clear with the composite material containing 80 wt % graphite, where the polar part of the surface tension increases from 0 mN/m (untreated) to 22.6 mN/m (after plasma treatment). It can also be seen that after plasma treatment, the increase in the polar part of the surface tension is more pronounced for all materials than after chemical treatment.

These results are very close to the previous work [23], where very similar values of surface tensions and their polar parts were measured for PP/EPDM-based composites filled with three different types of graphite (65 wt % or 80 wt %). 

### 3.5. Mechanical Properties of Adhesive Joint (Lap-Shear Test)

**Figure 7:**
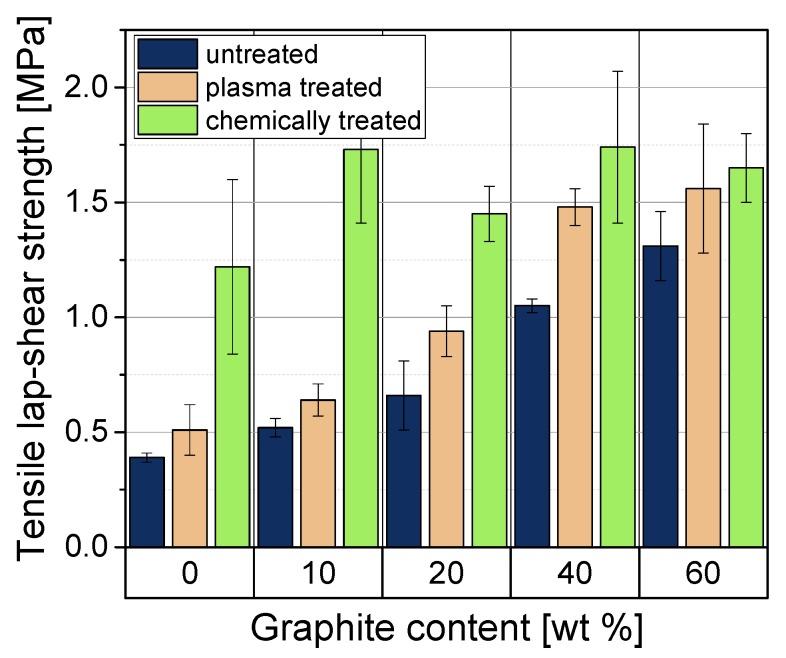
Tensile lap-shear strength of adhesively joined graphite composites.

The lap-shear test was used to determine the mechanical properties of adhesively joined PP/graphite composites. To assess the effect of surface pre-treatment on the mechanical properties of the joint, untreated, plasma and chemically treated samples were tested and compared. The tensile lap-shear strength of all specimens is shown in Figure 7. The tensile lap-shear strength of the adhesively joined samples increases with increasing graphite content in the substrate material. This can be attributed to a higher adhesion between adhesive and graphite on the surface of substrate materials. Another reason for this effect may be that with increasing stiffness of the substrate material (see Figure 1b) the maximum stresses in the adhesive layer are reduced. This effect of stress reduction can be deduced according to reference [29] from the elastostatic analysis of the adhesive bond (based on the Volkersen theory):

(4) $$\tau_{S,max}=\frac{F}{b}\cdot \sqrt{\frac{1}{2}\cdot \frac{1}{E_{sub}}\cdot \frac{G_{A}}{t_{A}}}$$

whereas *τ_S,max_* are maximal shear stresses in adhesive layer, *F* the force applied on both ends of the joint, *b* width of joined area, *E_sub_* the E-Modul of the substrate, *G_A_* the shear modulus of adhesive and *t_A_* the thickness of adhesive layer.

All untreated adhesively joined samples resulted in adhesive fracture.

After plasma treatment, a gradual increase in the tensile lap-shear strength with increasing filler content can be observed. This is due to the activation of the substrate surface by the attachment of reactive groups to the surface (e.g., –OH, –COOH, –CO groups) which produces more active and polar surface [30,31]. These reactive groups make it possible to produce stronger chemical bonds with the adhesive, which leads to the observed higher adhesion and higher tensile lap-shear strength. Most specimens fractured adhesively, with the exception of the 60 wt % graphite material where all specimens broke in the substrate. This means that the strength of the joint exceeds the strength of the substrate material itself. 

Chemical treatment with H_2_SO_4_ leads to a significant increase of tensile lap-shear strength, especially with PP and composites with lower filler contents (10 and 20 wt %). The values achieved are significantly higher than after plasma treatment. These samples fractured adhesively. For composites with higher filler contents (40 wt % and 60 wt %) the differences are not so pronounced compared to plasma treatment. Samples with higher filler contents fractured differently, either cohesively or in the substrate. The fact that after chemical treatment the tensile lap-shear strength is higher than after plasma treatment is caused not only by activation but also by etching of the surface, resulting in a rougher surface structure. The adhesive can fill microcavities on the etched, rougher surfaces, creating a stronger mechanical bond between the adhesive and substrate. The highest tensile lap-shear strength value of 1.74 ± 0.33 MPa was achieved with PP/40. For all chemical treated graphite composites, the tensile lap-shear strength remains more or less equal. These comparable values may be caused by etching the polypropylene on the surface of the specimens, resulting in a surface containing only graphite. Therefore, all graphite composites have a very similar surface structure after chemical treatment, resulting in similar adhesion between substrate and adhesive and comparable tensile lap-shear strengths values. Another effect is the roughness of the surface after chemical treatment, which with *R_a_* values of about 2 μm is larger than that of the non-chemically treated samples. The higher roughness leads to stronger mechanical bonds at the interface, which contributes to higher tensile lap-shear strength values for all materials. 

### 3.6. Surface Roughness

**Figure 8:**
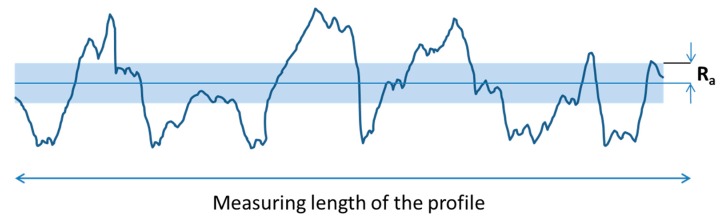
Graphical explanation of the *R_a_* profile factor.

**Figure 9:**
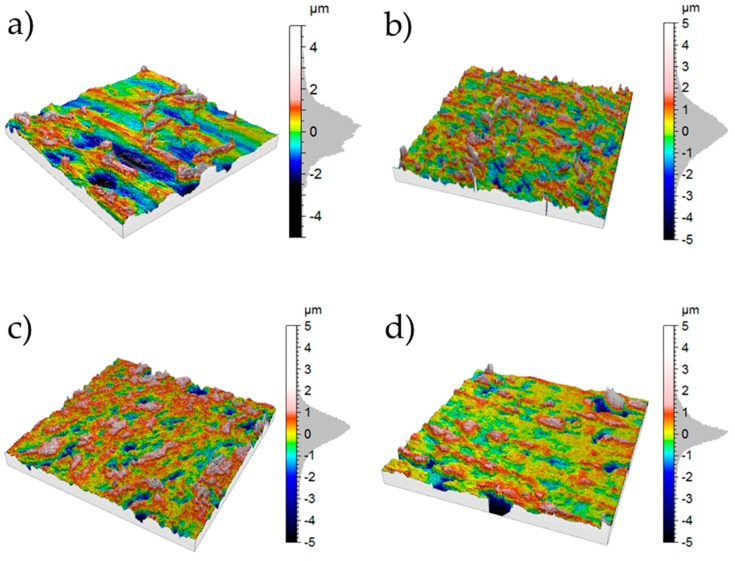
3D surface mapping of (**a**) pure PP; (**b**) PP/20; (**c**) PP/40 and (**d**) PP/80.

As mentioned before, the surface roughness of any substrate is also expected to have an influence on the adhesion of adhesively joined samples. Two factors were used to determine the surface roughness: the 2D profile factor *R_a_* expressed in µm and the 3D surface factor *S_dr_* expressed in %. The *R_a_* factor is calculated as arithmetical mean of the absolute height values within a scanning length and is used for the global evaluation of the roughness amplitude at a profile (Figure 8). *S_dr_* determines percentage difference between the real surface and the projected surface based on a 3D mapping. Table 4 shows the results of the surface roughness of injection molded graphite composite materials whose surface was milled. It was found that *R_a_* values of all materials remain at the same level as intended by the milling process. The *S_dr_* factors are highest for pure PP (15.3%) and lowest for composite materials with 40 wt % graphite (2.7%). Different values of the *S_dr_* factors may be caused by an inhomogeneous distribution of the graphite particles on the surface or by the milling process, in which graphite particles can be broken out of the composite surface. As visible in the 3D mapping in Figure 9, the surfaces of the composite materials look quite similar, with the exception of PP (Figure 9). On the pure PP surface, clear traces of the milling knife can be seen running across the entire surface.

For the untreated composites no correlation between the surface topology (roughness or 3D structure) and the tensile lap-shear strength values of adhesively joined PP/graphite composites can be found. The tensile lap-shear strength values increase with the graphite content (Figure 7), but the values for *R_a_* and *S_dr_* show no tendency at all. It can be concluded that the graphite content is the main influence on the shear strength values.

## 4. Conclusions

The present study showed that graphite-filled PP composites suitable for use as bipolar plates in fuel cells can be developed. The addition of expanded graphite to the PP matrix made it possible to adapt the property profile relevant to the application, such as electrical and thermal conductivity and mechanical stability. Composite materials with 80 wt % graphite showed the best suitability for bipolar plate applications of all the composite materials tested. PP/80 has a sufficiently high thermal conductivity (12.4 W/m·K). Although the electrical conductivity has increased significantly compared to PP and composites with lower graphite contents up to 15.9 S/cm, it is still below the required value (see Table 1). The PP/60 composite material achieved a flexural strength of 41.0 MPa, which meets the requirements for use in fuel cells (see Table 1). On the other hand, the tensile strength has decreased to 25.6 MPa compared to pure PP and is thus below the required values. 

As a further aspect, the possibility of adhesive joining of the bipolar plates as an alternative sealing method in the fuel cells was investigated. For this purpose, the surface tension of the composite materials was investigated. For a good wettability of the surface of the bipolar plate by the adhesive and a good adhesion between adhesive and substrate a high surface tension and polarity is necessary. It was found that with increasing filler content the surface tension also increases, but the polarities of all composites remain very low. Surface tension and polarity were significantly increased after surface pre-treatment (plasma and chemical), which was particularly remarkable for the polarities of graphite composites. Higher polarities were achieved after plasma treatment than after chemical treatment. Lap shear tests showed that the graphite composite materials developed can be successfully joined. The tensile lap-shear strength of adhesively joined composites increased significantly with increasing graphite content. Both surface pre-treatments had a positive effect on the joined composites, which led to higher tensile lap-shear strength. Surface pre-treatment led to a change in the type of failure from adhesive failure to cohesion or substrate failure (40 wt % and 60 wt % graphite composites). The results showed that chemical treatment is a better method than plasma treatment to improve the mechanical properties of adhesively joined graphite composites.

## Figures and Tables

**Table 1:** Overall requirements for bipolar plates in fuel cells [4].

Property	Value
Electrical conductivity	>100 S/cm
Thermal conductivity	>10 W/m·K
Tensile strength	>41 MPa
Flexural strength	>25 MPa

**Table 2:** Melt mixing process parameters of graphite composites.

Description	Graphite Content in Composite (Set Values) [wt %]	Graphite Content in Composite (TGA Values) [wt %]	Rotation Speed [rpm]	Torque [%]	Pressure at the Nozzle [bar]
PP	0	-	200	51	0
PP/10	10	10.6	200	50	0
PP/20	20	20.8	200	51	1
PP/40	40	40.1	200	57	9
PP/60	60	59.6	300	53	29
PP/80	80	78.7	300	74	119

**Table 3:** Values of the surface tension and its components of the liquids used for contact angle measurements [25].

Measuring Liquid	Total Surface Tension [mN/m]	Dispersive Part [mN/m]	Polar Part [mN/m]
Distilled water	72.8	21.8	51.0
1,5-pentanediol	43.3	27.6	15.7
Diiodmethane	50.8	50.8	0

**Table 4:** Surface roughness expressed using 2D profile and 3D surface factors.

Material	Surface Treatment	Profile	3D-Surface
		*R_a_* [µm]	*S_dr_* [%]
Pure PP	Milling	0.8	15.3
PP/10	Milling	0.7	7.3
PP/20	Milling	1.1	12.8
PP/40	Milling	0.5	2.7
PP/60	Milling	0.5	5.0
PP/80	Milling	0.5	10.0
